# Phenotypic Differences between Asian and African Lineage Zika Viruses in Human Neural Progenitor Cells

**DOI:** 10.1128/mSphere.00292-17

**Published:** 2017-07-26

**Authors:** Fatih Anfasa, Jurre Y. Siegers, Mark van der Kroeg, Noreen Mumtaz, V. Stalin Raj, Femke M. S. de Vrij, W. Widagdo, Gülsah Gabriel, Sara Salinas, Yannick Simonin, Chantal Reusken, Steven A. Kushner, Marion P. G. Koopmans, Bart Haagmans, Byron E. E. Martina, Debby van Riel

**Affiliations:** a Department of Viroscience, Erasmus MC, Rotterdam, The Netherlands; b Faculty of Medicine, Universitas Indonesia, Jakarta, Indonesia; c Department of Psychiatry, Erasmus MC, Rotterdam, The Netherlands; d Heinrich Pette Institute for Experimental Virology, Hamburg, Germany; e UMR1058, Pathogenesis and Control of Chronic Infections, INSERM, Université de Montpellier, Etablissement Français Du Sang, Montpellier, France; f Artemis One Health Research Foundation, Utrecht, The Netherlands; University of Michigan—Ann Arbor

**Keywords:** African strains, Asian strains, growth kinetics, human neural progenitor cells, neuronal cells, one-step growth curve, pathogenesis, phenotype, Zika virus, cell death

## Abstract

Recent Zika virus (ZIKV) infections have been associated with a range of neurological complications, in particular congenital microcephaly. Human neural progenitor cells (hNPCs) are thought to play an important role in the pathogenesis of microcephaly, and experimental ZIKV infection of hNPCs has been shown to induce cell death. However, the infection efficiency and rate of cell death have varied between studies, which might be related to intrinsic differences between African and Asian lineage ZIKV strains. Therefore, we determined the replication kinetics, including infection efficiency, burst size, and ability to induce cell death, of two Asian and two African ZIKV strains. African ZIKV strains replicated to higher titers in Vero cells, human glioblastoma (U87MG) cells, human neuroblastoma (SK-N-SH) cells, and hNPCs than Asian ZIKV strains. Furthermore, infection with Asian ZIKV strains did not result in significant cell death early after infection, whereas infection with African ZIKV strains resulted in high percentages of cell death in hNPCs. The differences between African and Asian lineage ZIKV strains highlight the importance of including relevant ZIKV strains to study the pathogenesis of congenital microcephaly and caution against extrapolation of experimental data obtained using historical African ZIKV strains to the current outbreak. Finally, the fact that Asian ZIKV strains infect only a minority of cells with a relatively low burst size together with the lack of early cell death induction might contribute to its ability to cause chronic infections within the central nervous system (CNS).

**IMPORTANCE** The mechanism by which ZIKV causes a range of neurological complications, especially congenital microcephaly, is not well understood. The fact that congenital microcephaly is associated with Asian lineage ZIKV strains raises the question of why this was not discovered earlier. One possible explanation is that Asian and African ZIKV strains differ in their abilities to infect cells of the CNS and to cause neurodevelopmental problems. Here, we show that Asian ZIKV strains infect and induce cell death in human neural progenitor cells—which are important target cells in the development of congenital microcephaly—less efficiently than African ZIKV strains. These features of Asian ZIKV strains likely contribute to their ability to cause chronic infections, often observed in congenital microcephaly cases. It is therefore likely that phenotypic differences between ZIKV strains could be, at least in part, responsible for the ability of Asian ZIKV strains to cause congenital microcephaly.

## Abstract (precis)

The mechanism by which ZIKV causes a range of neurological complications, especially congenital microcephaly, is not well understood. The fact that congenital microcephaly is associated with Asian lineage ZIKV strains raises the question of why this was not discovered earlier. One possible explanation is that Asian and African ZIKV strains differ in their abilities to infect cells of the CNS and to cause neurodevelopmental problems. Here, we show that Asian ZIKV strains infect and induce cell death in human neural progenitor cells—which are important target cells in the development of congenital microcephaly—less efficiently than African ZIKV strains. These features of Asian ZIKV strains likely contribute to their ability to cause chronic infections, often observed in congenital microcephaly cases. It is therefore likely that phenotypic differences between ZIKV strains could be, at least in part, responsible for the ability of Asian ZIKV strains to cause congenital microcephaly.

## INTRODUCTION

Since the emergence of Zika virus (ZIKV) in 2015 in South America, infections have caused a wide spectrum of neurological diseases, such as Guillain-Barré syndrome, myelitis, meningoencephalitis, and in particular congenital microcephaly (1). Even though ZIKV was first detected in Uganda in 1947 in a rhesus monkey and has caused repeated outbreaks since 2007, not much was known about the pathogenesis of disease caused by ZIKV before the 2015 outbreak. Since then, several studies have shown that ZIKV can infect a variety of neuronal cells, but more insight into the pathogenesis of ZIKV-induced central nervous system (CNS) diseases is needed (2).

An important question that remains is whether the emergence of ZIKV in South America and the associated clinical findings are the result of genetic and phenotypic changes in the emerging ZIKV strain or whether they can be attributed to the introduction of ZIKV in a large naive population (3). Phylogenetically, two distinct lineages of ZIKV exist: the African lineage and the Asian lineage (4). The current outbreak strain belongs to the Asian lineage, and sequence analysis revealed that the virus has changed significantly over the last 50 years, both in nucleotide sequences and amino acid composition (4, 5). The prototype ancestral ZIKV strain, MR766, of the African lineage has been used in many initial studies (6–9), but recent *in vitro* and *in vivo* studies have shown some differences between African and Asian ZIKV strains (10–14). Whether there are also phenotypic differences between Asian ZIKV strains, caused by amino acid substitutions acquired just before the outbreak in South America, is currently unknown (5).

ZIKV has been shown to replicate and induce cell death in neuronal cells of fetal mice (5, 15), as well as in human neural progenitor cells and brain organoids (6, 7, 11, 12), a mechanism thought to play an important role in the pathogenesis of ZIKV-induced microcephaly. A recent study has shown that an African ZIKV strain might be able to infect human neural stem cells (hNSCs) and astrocytes more efficiently than Asian ZIKV strains (12). However, a comprehensive study on the replication kinetics and the ability to cause cell death of different African and Asian ZIKV strains is currently lacking (2).

To be able to detect phenotypic differences between Asian and African ZIKV strains or between recent Asian ZIKV strains, it is important to characterize and understand the *in vitro* replication kinetics—including the infection efficiency, burst size, and ability to cause cell death—of these viruses. Therefore, we determined the replication kinetics of two Asian ZIKV strains (isolated in 2013 and 2016) and two African ZIKV strains (isolated in 1947 and 1961) on induced pluripotent stem cell-derived human neural progenitor cells (hNPCs) and several human neural cell lines.

## RESULTS

### Phylogenetic and amino acid variance analysis of ZIKV strains selected in this study.

**FIG 1:**
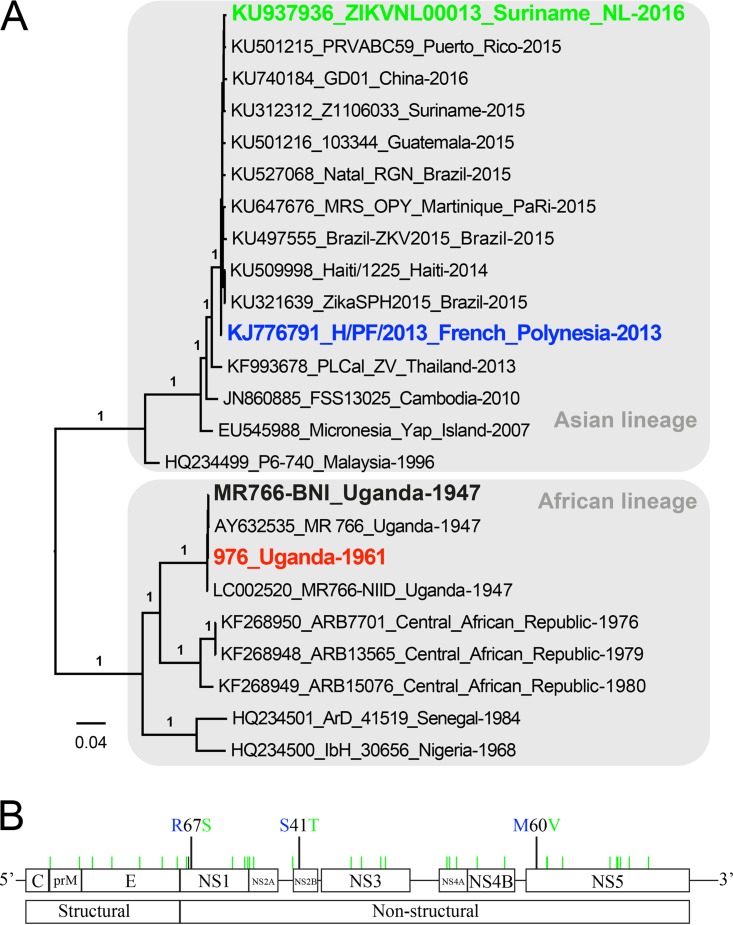
Phylogenetic analysis of ZIKV strains used in this study and genomic organization and mutations between the Asian lineage ZIKV strains. (A) Nucleotide sequences of representative Zika virus genomes were analyzed, and a phylogenetic tree was constructed using the PhyML method. Values at branches show the result of the approximate likelihood ratio; values of <0.70 are not shown. (B) Genome organization and mutations between Asian lineage H/PF/2013 (ZIKV^AS-FP13^) and ZIKVNL00013 (ZIKV^AS-Sur16^) ZIKV strains.

Four ZIKV strains were included in this study (Fig. 1A). Two African strains, ZIKV MR766 (ZIKV^AF-MR766^) and Uganda 976 (ZIKV^AF-976^), were isolated in 1947 and 1961, respectively, and passaged on mouse brain tissue and Vero cells. The two Asian ZIKV strains included were H/PF/2013 (ZIKV^AS-FP13^) and ZIKVNL00013 (ZIKV^AS-Sur16^), which were isolated in 2013 and 2016, respectively, and passaged 4 times on Vero cells. A phylogenetic analysis of the complete genome of the selected strains with other ZIKV genomes shows their positions in the Asian or African lineages (Fig. 1A). There are over 50 amino acid (aa) differences between the African and Asian ZIKV strains that have previously been described (5). The amino acid differences between the Asian ZIKV strains were located in the NS1 (R67S; position 863), NS2B (S41T; position 1417), and NS5 (M60V; position 2634) proteins (Fig. 1B). Of these amino acid differences, the mutation at position 2634 is only observed in viruses isolated from the recent outbreak (4, 5, 16). The amino acid difference at position 1417 of ZIKV^AS-Sur16^ was not present in the original clinical isolate but was acquired during passaging on Vero cells (17).

### Growth curves of Asian and African ZIKV strains on neuronal cells.

**FIG 2:**
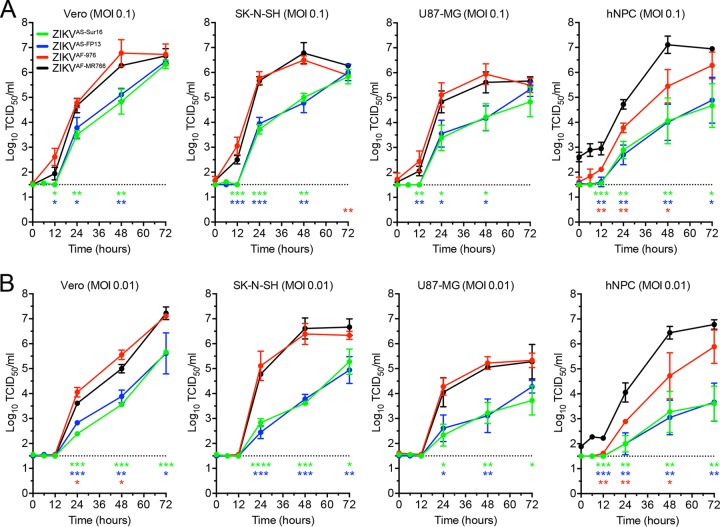
Growth curves of ZIKV strains on Vero, SK-N-SH, and U87-MG cells and hNPCs. (A and B) Growth curves of Asian lineage strains H/PF/2013 (ZIKV^AS-FP13^ [blue lines]) and ZIKVNL00013 (ZIKV^AS-Sur16^ [green lines]) and African lineage MR766 (ZIKV^AF-MR766^ [black lines]) and 976 Uganda (ZIKV^AF-976^ [red lines]) on Vero, human neuroblastoma (SK-N-SH), and human glioblastoma (U87-MG) cells and human neuronal progenitor cells (hNPCs) at MOI of 0.1 (A) and 0.01 (B). Data are presented as means with standard deviations from at least 3 independent experiments. Statistical significance was calculated using the Student *t* test in comparison with ZIKV^AF-MR766^. *, *P* ≤ 0.05; **, *P* ≤ 0.01; ***, *P* ≤ 0.001; ****, *P* ≤ 0.0001. TCID_50_, 50% tissue culture infectious dose.

Growth curves were determined for ZIKV^AS-FP13^, ZIKV^AS-Sur16^, ZIKV^AF-MR766^, and ZIKV^AF-976^ by *in vitro* infections using low multiplicities of infection (MOI [0.1 and 0.01]) on SK-N-SH cells (human neuroblastoma cells), U87-MG cells (human glioblastoma cells), Vero cells, and hNPCs. Growth curves showed that all cells supported replication of all four ZIKV strains included, but virus titers were significantly lower for both Asian strains compared to ZIKV^AF-MR766^ strains on cells of the Vero, SK-N-SH, and U87-MG lines and hNPCs (Fig. 2A and B). On hNPCs, ZIKV^AF-MR766^ grew faster to significantly higher titers than ZIKV^AF-976^ (Fig. 2A and B). There were no evident differences in the growth curves between ZIKV^AS-FP13^ and ZIKV^AS-Sur16^.

### One-step growth curves of Asian and African ZIKV strains on neuronal cells.

**FIG 3:**
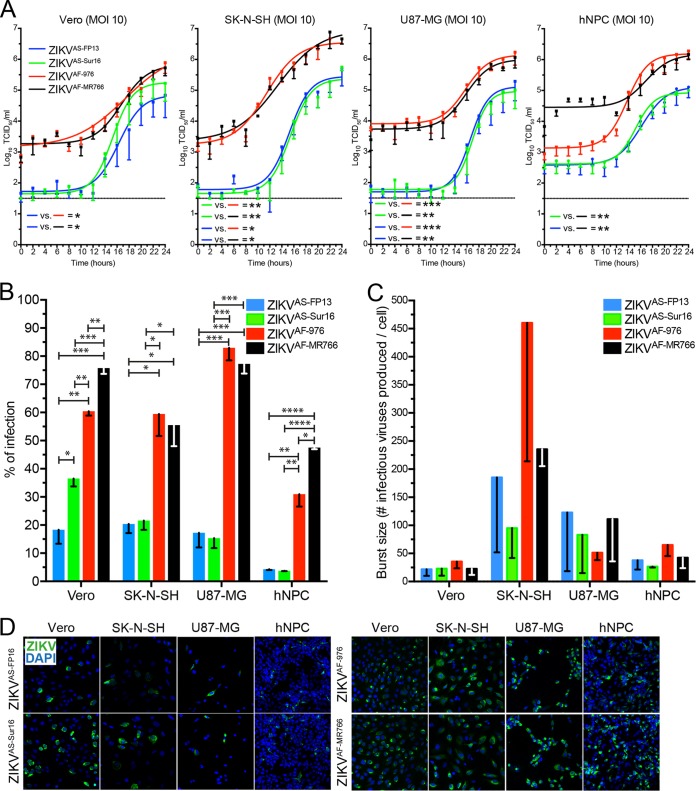
One-step growth curve (OSGC) kinetics of Asian and African lineage ZIKV strains. (A) OSGCs of Asian lineage strains H/PF/2013 (ZIKV^AS-FP13^ [blue lines]) and ZIKVNL00013 (ZIKV^AS-Sur16^ [green lines]) and African lineage MR766 (ZIKV^AF-MR766^ [black lines]) and 976 Uganda (ZIKV^AF-976^ [red lines]) on Vero, human neuroblastoma (SK-N-SH), and human glioblastoma (U87-MG) cells and human neuronal progenitor cells (hNPCs). (B) Percentage of ZIKV infection determined by immunofluorescent microscopy of two Asian and two African ZIKV strains. (C) Number of infectious viruses produced per cell (burst size) for each virus in the 4 different cell lines. (D) Representative immunofluorescent images of ZIKV-infected cells stained for ZIKV antigen (green). Magnification, ×200. For panels A and B, data are presented as means with standard deviations and nonlinear curve fit for at least 3 independent experiments. For panel C, data are presented as means with standard errors of the means from at least 3 independent experiments. Statistical significance was calculated using a one-way ANOVA with Tukey’s multiple comparisons test for panel A. For panels B and C, the Student *t* test was used. *, *P* ≤ 0.05; **, *P* ≤ 0.01; ***, *P* ≤ 0.001; ****, *P* ≤ 0.0001. TCID_50_, 50% tissue culture infectious dose.

One-step growth curves (OSGCs) were assessed *in vitro* by using a high MOI (MOI of 10). Data from OSGC experiments on the different cell lines were used to calculate the percentage of infection and burst size (progeny virus produced per cell). OSGCs showed that baseline virus titers were higher for the African ZIKV strains than for the Asian ZIKV strains, and both African ZIKV strains grew to higher titers on all cells (Fig. 3A). In all cells, African ZIKV strains infected more cells than the Asian ZIKV strains (Fig. 3B and D). The numbers of virus particles produced did not differ significantly between the different ZIKV strains. However, there was a trend toward burst size being higher in SK-N-SH cells (~100 to 400 infectious virus particles/cell) than in Vero cells (~20 to 40 infectious virus particles/cell), U87-MG cells (~50 to 150 infectious virus particles/cell), and hNPCs (~40 to 75 infectious virus particles/cell) (Fig. 3C).

### Induction of cell death by Asian and African ZIKV strains.

The ability of ZIKV^AS-Sur16^, ZIKV^AS-FP13^, and ZIKV^AF-MR766^ to cause cell death in hNPCs at 24, 48, and 72 hours postinfection (hpi) was determined after infection with an MOI of 3. Cells were stained for either ZIKV antigen or terminal deoxynucleotidyltransferase-mediated dUTP-biotin nick end labeling (TUNEL [DNA fragmentation]) and measured by flow cytometry. Uninfected cells and β-propiolactone (BPL)-inactivated ZIKV^AF-MR766^ were included as controls. In addition, cells were fixed at 48 hpi for immunofluorescent double staining for ZIKV antigen and TUNEL. A maximum of 12% TUNEL positivity was observed in BPL control and negative-control cells 72 hpi.

**FIG 4:**
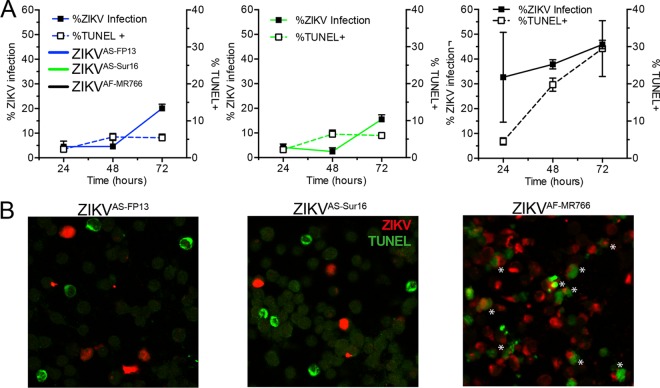
Ability to cause cell death of African and Asian lineage ZIKV strains in human neural progenitor cells. (A) Percentage of human neural progenitor cells infected with African lineage ZIKV strain ZIKV^AF-MR766^ (black lines) and Asian lineage ZIKV strains H/PF/2013 (ZIKV^AS-FP13^ [blue lines]) and ZIKVNL00013 (ZIKV^AS-Sur16^ [green lines]) and percentage of TUNEL-positive cells measured over 72 h. The left *y* axis represents the percentage of cells infected with ZIKV, and the right *y* axis represents the percentage of TUNEL-positive cells. Data are presented as means with standard errors of the means from at least 3 independent experiments. (B) Representative immunofluorescent images of human neural progenitor cells infected with different ZIKV strains 48 h postinfection and double stained for ZIKV antigen (red) and TUNEL (green). Asterisks indicate double-positive cells. Magnification, ×200.

Infection with ZIKV^AS-FP13^ and ZIKV^AS-Sur16^ resulted in approximately 20% infection at 72 hpi, and up to 9% of cells were TUNEL positive, the latter comparable to control and BPL-treated cells. Immunofluorescent staining revealed that very few TUNEL-positive cells were ZIKV infected (Fig. 4A and B). In contrast, infection with ZIKV^AF-MR766^ resulted in 46% infection and 30% TUNEL-positive cells at 72 hpi (Fig. 4A). Immunofluorescent staining revealed that in ZIKV^AF-MR766^-infected cells, the majority of TUNEL-positive cells were also infected, indicating that ZIKV^AF-MR766^ is able to induce cell death early after infection in hNPCs (Fig. 4B).

## DISCUSSION

This study on the *in vitro* replication of different ZIKV strains shows that African ZIKV strains replicated more efficiently in Vero, human glioblastoma, and human neuroblastoma cells and hNPCs than Asian ZIKV strains. In hNPCs, which are considered an important target cell type for the development of congenital microcephaly, African ZIKV strains induced cell death early after infection, which was not observed after infection with Asian ZIKV strains.

Overall there were few phenotypic differences between ZIKV^AS-FP13^ and ZIKV^AS-Sur16^. This suggests that the mutations between these viruses, including position 2634 unique for ZIKV isolated from this outbreak, does not lead to large phenotypic changes, at least not in these cell lines. The fact that Vero and SK-N-SH cells permit efficient replication of Asian ZIKV strains supports the usage of these cells for virus isolation from clinical samples (18).

The replication kinetics and ability to cause cell death in hNPCs differed substantially between African and Asian ZIKV strains. Asian ZIKV strains infect and replicate less efficiently in hNPCs than the African ZIKV strains. This “reduced” replication is not an intrinsic feature of Asian ZIKV strains, since they replicate to high titers in Vero and SK-N-SH cells. One possible explanation for the increased ability of African ZIKV strains to infect hNPCs in this study could be that these strains have adapted to neural cells due to their passage history in mouse brain tissues (18) and that the 4-aa deletion in the E protein of these viruses contributes to the observed phenotype. However, similar results—high percentage of infection and induction of cell death in hNPCs—have been observed with a low-passage-number 1989 African ZIKV strain (ArB41644) (12). Upon sequencing, we did not find any deletion in the E protein (GenBank accession no. KY576904) of this low-passage-number African lineage ZIKV strain. Therefore, these studies together suggest that Asian ZIKV strains infect hNPCs less efficiently than African ZIKV strains, regardless of the passage history of the ZIKV strains. Both Asian lineage ZIKV strains do not seem to induce cell death early after infection, whereas ZIKV^AF-MR766^ does. This fits with previous observations, where more apoptotic nuclei were observed after infection with an African ZIKV strain than with an Asian ZIKV strain (12), which suggests that there are intrinsic differences between Asian and African ZIKV strains in their ability to cause cell death in hNPCs.

The observed phenotypic characteristics of Asian lineage ZIKV strains might contribute to their ability to cause chronic infection in tissues of the CNS (17–21). First, Asian ZIKV strains infect relatively few hNPCs. Second, Asian ZIKV strains release less than 40 infectious virus particles per infected hNPC, which is relatively low compared to other viruses, such as influenza virus and simian immunodeficiency virus (SIV) (22, 23). A low burst size has previously also been associated with prolonged virus replication within the CNS for Japanese encephalitis virus, another flavivirus (24). Finally, Asian ZIKV strains do not seem to induce cell death early after infection in neural progenitor cells, which might result in chronic infection and replication within the CNS (19, 20). This fits with a recent animal study using Stat2^−/−^ mice, which showed that African ZIKV strains induce short episodes of severe neurological symptoms followed by lethality, while Asian ZIKV strains manifest prolonged signs of neuronal malfunctions. Limited mortality was also only observed in one Asian ZIKV strain (25).

Taken together, we here show that African and Asian ZIKV strains differ in their abilities to infect and replicate in different neural cells, as well as their abilities to cause cell death early after infection. This implies that caution is necessary against extrapolation of experimental data obtained using historical African ZIKV strains to the current outbreak. In addition, the fact that Asian ZIKV strains infect only a minority of cells with a relatively low burst size together with the lack of early cell death induction might contribute to their ability to cause chronic infections within the CNS.

## MATERIALS AND METHODS

### Cells.

Human induced pluripotent stem cell (IPSC)-derived neural progenitor cells (NPCs) (Ax0015; Axol, Cambridge, United Kingdom) were cultured in neural maintenance basal medium with supplements (Ax0031; Axol) according to the manufacturer’s specification. Human IPSC-derived NPCs were grown on plates coated with 20 µg/ml laminin (L2020; Sigma-Aldrich). Human neuroblastoma SK-N-SH and human glioblastoma U87-MG cells were purchased from Sigma-Aldrich and grown in Eagle’s minimum essential medium (EMEM) with Earle’s balanced salt solution (EBSS [Lonza, Breda, The Netherlands) containing 10% heat-inactivated fetal bovine serum (HI-FBS [Lonza]), 100 U penicillin (Gibco Life Sciences, USA), 100 µg/ml streptomycin (Gibco), 2 mM l-glutamine (Lonza), 1% nonessential amino acids (Lonza), 1 mM sodium pyruvate (Gibco), and 1.5 mg/ml sodium bicarbonate (Lonza). Both immortalized cell lines SK-N-SH and U87-MG were used below passage 25. Vero cells (ATCC, USA) were grown in Dulbecco’s modified Eagle’s medium (DMEM) containing 10% HI-FBS, 100 µg/ml streptomycin, 100 U penicillin, 2 mM l-glutamine, 1% sodium bicarbonate, and 1% HEPES buffer (all from Gibco). Human NPCs are primary cells, while the other cells are from immortalized cell lines. All cells used in this study tested negative for *Mycoplasma* sp.

### Viruses.

Zika virus strain Uganda 976 (ZIKV^AF-976^) was provided by Misa Korva (University of Ljubljana; European Virus Archive goes Global [EVAg] no. 007V-EVAg1585). Zika virus MR766 (ZIKV^AF-MR766^) was provided by Stephan Günther (Bernhard-Nocht-Istitute for Tropical Medicine). This strain has three nucleotides different (C6258T, G6273T, and G10671A) from the reference MR766 strain (GenBank accession no. KU955594). Zika virus strain H/PF/2013 (ZIKV^AS-FP13^) was obtained from UMR 190-Unite Des Virus Emergents (EVAg no. 001V-EVA1545). Zika virus Suriname ZIKVNL00013 (ZIKV^AS-Sur16^) was isolated from a patient in The Netherlands (EVAg no. 011V-01621) (17). All virus stocks used in this study were grown in Vero cells. The following passage numbers were used: passage 6 (P6) for ZIKV^AF-976^, unknown for ZIKV^AF-MR766^, and P4 for ZIKV^AS-FP13^ and ZIKV^AS-Sur16^. Virus titers were determined in Vero cells 5 days after infection by means of cytopathic effect (CPE), and the 50% tissue culture infective dose (TCID_50_) was calculated using the Spearman-Kärber method (26). All virus stocks were stored at −80°C until further use. A summary of the isolation history of all ZIKV strains used in this study and related information is provided in Table 1.

**TABLE 1:** Source host, isolation, and passage history as well as GenBank accession numbers of the ZIKV strains used in the study^a^

Lineage	Strain	Source host	Yr of isolation	Location	Passage history	GenBank accession no.	EVAg no.
Asian	ZIKVNL00013	Human	2016	Suriname	4× on Vero cells	KU937936	011V-01621
	H/PF/2013	Human	2013	French Polynesia	4× on Vero cells	KJ776791.2	001V-EVA1545
African	MR766	Monkey	1947	Uganda	Unknown (multiple times on SMB and 1× on Vero cells)	KU955594	NA
	Uganda 976	Monkey	1961	Uganda	2× on SMB, 3× on Vero E6 cells, 1× on Vero cells	NA	007V-EVAg1585

a Abbreviations: SMB, suckling mouse brain; Vero, African green monkey kidney cells; Vero E6, African green monkey kidney clone E6 cells; EVAg, European Virus Archive goes Global; NA, not available.

### Next-generation sequencing.

For genomic characterization of the virus strains, RNA was isolated from 140 μl of the virus stocks with the QIAmp Viral Mini RNA kit (Qiagen, Germany). Subsequently, the product was eluted in 40 μl double-distilled water. Viral metagenomic libraries were constructed with 454 pyrosequencing as previously described (27), and the libraries were sequenced using a 454 GS-Junior machine (Roche, USA) according to the manufacturer’s instructions.

### Phylogenetic analysis.

Nearly full-length ZIKV genomes of 4 isolates (ZIKV^AF-976^, ZIKV^AF-MR766^, ZIKV^AS-FP13^, and ZIKV^AS-Sur16^) and other reference sequences were obtained from the GenBank database. The sequences were aligned using ClustalW, and a phylogenetic tree was constructed by using the PhyML method in SeaView 4 (http://pbil.univ-lyon1.fr/software/seaview) with the approximate likelihood ratio test based on a Shimodaira-Hasegawa-like procedure which used general time reversible as a substitution model. Nearest-neighbor interchange, subtree pruning, and regrafting-based tree search algorithms were used to estimate tree topologies (28). The obtained tree was visualized by using FigTree version 1.3.1 (http://tree.bio.ed.ac.uk/software/figtree).

### Replication kinetics of Zika virus strains.

Replication kinetics of ZIKV strains ZIKV^AS-976^, ZIKV^AS-MR766^, ZIKV^AS-FP13^, and ZIKV^AS-Sur16^ were studied *in vitro* by means of one-step growth curve (OSGC) experiments with a multiplicity of infection (MOI) of 10 and focal experiments (growth curves) with MOI of 0.1 and 0.01. Human neural progenitor cells and SK-N-SH, U87MG, and Vero cells were seeded into 96-well plates (2 × 10^4^ cells) (Greiner, USA). After 24 h, monolayers were inoculated with the different ZIKV strains or Vero cell culture medium as a control at an MOI of 10, 0.1, or 0.01 for 1 h at 37°C in 5% CO_2_. After 1 h of virus absorption, the inoculum was removed and cells were washed 3 times and replenished with fresh medium that contains 2% FCS (no FCS for hNPCs) and cultured for 24 or 72 h at 37°C for the OSGC and growth curve, respectively. For the OSGC, supernatant was collected every 2 h up to 24 h and subsequently stored at −80°C until virus titer determination. Cells were fixed in 4% paraformaldehyde (PFA) for 20 min at room temperature, washed with phosphate-buffered saline (PBS), and permeabilized and stored in 70% ethanol for immunofluorescent staining. For the growth curves, supernatant was collected at time points 0, 1, 12, 24, 48, and 72 hpi and stored at −80 until use. All growth curves and OSGCs were performed 3 times (biological replicates), and each growth curve included duplicate (technical replicates) measurements from which the average was used for future analysis.

### Determination of virus titers.

Virus titers (TCID_50_) in the supernatant were determined by endpoint titrations on Vero cells. Tenfold serial dilutions were made and inoculated onto a monolayer of Vero cells. Cytopathic effect (CPE) was determined at 5 days postinfection (dpi), and virus titers were calculated using the Spearman-Kärber method (26). An initial 1:10 dilution of supernatant resulted in a detection limit of 10^1.5^ TCID_50_/ml.

### Immunofluorescence microscopy.

Infected cells from the OSGC at the time when 50% of virus particles are released (BT_50_) were fixed with 4% PFA for 20 min at room temperature, washed, and permeabilized with 70% ethanol. Subsequently, cells were washed twice in PBS and incubated for 1 h in the dark and at room temperature with anti-*Flavivirus* group antigen (MAB10216, clone D1-4G2-4-15, 1:200 dilution; Millipore, Germany) or mouse IgG2a isotype control (MAB003, 1:50 dilution; R&D Systems) in PBS containing 0.1% bovine serum albumin (BSA). Afterward, the cells were washed three times with PBS–0.1% BSA and incubated for 1 h with goat anti-mouse IgG2a conjugated with Alexa Fluor 488 (1:250 dilution; Life Technologies, Inc., The Netherlands) in PBS–0.1% BSA at room temperature and in the dark. After 1 h, cells were washed three times and mounted with ProLong Diamond Antifade mountant with DAPI (4′,6-diamidino-2-phenylindole [Life Technologies, Inc., USA]). Zika virus-infected cells were identified by use of a Zeiss LSM 700 confocal laser scanning microscope fitted on an Axio observer Z1 inverted microscope (Zeiss). All images were processed using Zen 2010 software (Zeiss). Per sample, 5 high-power fields were photographed and scored blindly by three individuals to determine the percentages of infected and noninfected cells.

### Calculation of percentage of infection and burst size.

The percentage of infection and burst size were calculated from the OSGC experiment. The burst size is defined as the number of progeny virus particles produced per infected cell and was calculated as follows. The time at which half the number of progeny virus were released into the supernatant (50% effective concentration [EC_50_] for dose-response curve) was determined, which was calculated by using a nonlinear regression analysis (sigmoidal dose-response, variable slope) in GraphPad Prism 6.0h using the infectious virus titer data from the OSGC measured over 24 h (2-h increments). At this time point, infected cells were fixed and stained for ZIKV (as described above), and the number of infected cells was calculated by counting virus-infected/uninfected cells in 5 randomly chosen panels in duplicate by 3 blind assessors. The average number of infected cells from 5 panels was taken and corrected for the surface area of a single 96-well flat bottom plate. Next, the infectious virus titer over 24 h was calculated by subtracting time point 0 from 24 h, which then was divided by the number of infected cells, resulting in the number of progeny virus particles produced per infected cell.

### TUNEL assay.

Human neural progenitor cells (hNPCs) were cultured in a 24-well plate and inoculated with ZIKV^AS-MR766^, ZIKV^AS-FP13^, or ZIKV^AS-Sur16^ at an MOI of 3. In addition, ZIKV^AS-MR766^ was inactivated using β-propiolactone (BPL) (1:4,000 vol/vol; Sigma-Aldrich, USA) at 4°C for 48 to 72 h. Subsequently, BPL was inactivated for 24 h at 37°C. Both inactivated ZIKV^AS-MR766^ and Vero cell culture supernatant served as negative controls. Viruses and controls were allowed to absorb for 1 h, after which hNPCs were washed three times in hNPC medium. Subsequently the medium was replenished with fresh medium and cultured at 37°C for 24, 48, or 72 h. The numbers of dead cells were measured with a Sigma-Aldrich *In situ* cell death detection kit with fluorescein (Sigma-Aldrich, USA). Briefly, the cells were first fixed with 4% PFA and permeabilized with a 1:1 dilution of 1% Triton X-100 and 70% ethanol. Noninfected cells were treated with 180 IU/ml DNase (Roche Diagnostics, Mannheim, Germany) for 15 min at room temperature to serve as a positive control. The *In situ* cell death detection kit with fluorescein was used according to the manufacturer’s instructions. Cells stained only with labeling solution were used as a negative control as suggested by the manufacturer. The number of TUNEL-positive cells was measured using a BD FACSCanto II (BD Biosciences, USA). Data were analyzed using FlowJo 10 software (Ashland, OR, USA). All experiments were performed three times (biological replicates), and each experiment included duplicate (technical replicate) measurements from which the average was calculated and used for further analysis.

### Flow cytometry assay.

Cells were infected the same way as described for the TUNEL assay. At time points 24, 48, and 72 h, cells were collected, fixed, and permeabilized using BD Cytofix/Cytoperm solution (BD Biosciences, USA) according to the manufacturer’s instructions. Cells were blocked using 10% normal goat serum (NGS [Dako, Denmark]) for 10 min on ice. Subsequently, Zika virus was detected using mouse monoclonal antibody against anti-flavivirus group antigen (MAB10216, clone D1-4G2-4-15; Millipore, Germany) at a 1:200 dilution or mouse IgG2a isotype control (MAB003; Dako, Denmark) at a 1:50 dilution in BD Perm/Wash containing 2% NGS and incubated for 1 h on ice and in the dark. Cells were washed twice, and goat anti-mouse IgG2a conjugated with Alexa Fluor 488 (Life Technologies, Inc., The Netherlands) at a 1:250 dilution was incubated for 1 h in the dark and on ice. After incubation of the secondary antibody, cells were washed twice and resuspended in fluorescence-activated cell sorter (FACS) buffer. The percentage of infected cells was measured using a BD FACSCanto II (BD Biosciences, USA). Data were analyzed using FlowJo 10 software (Ashland, OR, USA). All experiments were performed three times (biological replicates), and each experiment included duplicate (technical replicate) measurements from which the average was calculated and used for further analysis.

### Statistical analysis.

The statistical analyses were performed using GraphPad Prism 6.0h software (La Jolla, CA) for Mac. Student’s *t* test was used for comparison between two groups. For comparison between multiple groups, one-way analysis of variance (ANOVA) with Tukey’s multiple-comparison test was used. *P* values of ≤0.05 were considered significant.

### Accession number(s).

Sequences of the E protein from African ZIKV strain ArB41644 have been submitted to GenBank under accession no. KY576904.
